# Preferential Geographic Distribution Pattern of Abiotic Stress Tolerant Rice

**DOI:** 10.1186/s12284-018-0202-9

**Published:** 2018-02-08

**Authors:** A N M Rubaiyath Bin Rahman, Jianhua Zhang

**Affiliations:** 1 Department of Biology, Hong Kong Baptist University, Hong Kong, China; 2 State Key Laboratory of Agrobiotechnology, The Chinese University of Hong Kong, Shatin, Hong Kong China

**Keywords:** Abiotic Stress Tolerance, Alkali Tolerance, Cold Tolerance, Drought Tolerance, Flood Tolerance, Genebank Accession, Geographic Distribution, Salt Tolerance, Bangladesh

## Abstract

Crop productivity and stability of the food system are threatened by climate change, mainly through the effects of predicted abiotic stresses. Despite extensive research on abiotic stress tolerance in the past decades, the successful translation of these research to fields/farmers is scarce. The impelling demand of climate resilient varieties, and the poor translation of research into the field despite the availability of high throughput technologies lead us to critically analyse a neglected aspect of current abiotic stress tolerance research. Although environmental factors play the most important role in the development of adaptive traits of plants, most abiotic stress tolerance research ignores eco-geographic aspects of highly stress tolerant accessions. In this review, we critically examined the geographic distribution pattern of highly tolerant rice accessions of all major abiotic stresses along with one micronutrient deficiency. Remarkably, we identified a shared geographic distribution pattern of highly tolerant accessions for all abiotic stresses including zinc deficiency despite the sparseness of highly tolerant accessions. The majority of these tolerant accessions predominately originated from Bangladesh centred narrow geographic region. We therefore analysed the climatic and agro-ecological features of Bangladesh. Considering the threat of climate change on global food security and poverty, urgent concerted research efforts are necessary for the development of climate resilient rice varieties utilizing the technological advancement, know-hows, and the preferential distribution pattern of abiotic stress tolerant rice.

**Electronic supplementary material:**

The online version of this article (10.1186/s12284-018-0202-9) contains supplementary material, which is available to authorized users.

## Background

Poverty, food insecurity, and climate change are the three prime global challenges. Considering their impacts, all three were chosen for the 2030 agenda for sustainable development goals by the United Nations (UN General Assembly, 2015). However, among these three, the interrelation between climate change and food security, and more specifically agricultural production are well documented (Schmidhuber and Tubiello 2007; Lobell et al. 2008; Wheeler and von Braun 2013; Brown et al. 2015). All climate-modelling studies predict that climate change is likely to change precipitation patterns (resulting in more drought or floods), rise of temperature (heat stress) and sea levels (flood and saline intrusion), occurrence of more frequent and severe weather extremes (drought/flood/cyclone etc.). Weather extremes significantly reduce crop production, and can even destroy complete crop production in severe cases. It has been estimated that unfavourable climatic conditions and inappropriate soil can account for over 70% of the yield loss of major crops (Boyer 1982).

Rice is the single most important primary food source crop for half of the world’s population (GRiSP 2013). Nearly all rice is produced (90%) and consumed (87%) in Asia. Overall, rice accounts for nearly 30% of the calorie demands for more than 3 billion Asians. However, in many rice-consuming countries like Bangladesh, Cambodia, Indonesia, and Vietnam, rice makes up for 45-70% of the calorie requirements (GRiSP 2013). Nevertheless, the majority of the population in rice-producing areas, particularly in numerous Asian and African countries, still suffer from hunger, malnutrition and extreme poverty. It was projected that global rice production need to be doubled by 2030 to cope with the impending population demand. However, although rice production has increased over three-fold in the last 4 decades, the growth rate of rice yield is far below the required projected demand (Ray et al. 2013). Moreover, the impact of climate change on agriculture (IMPACT modelling) predicts that global rice production will decline by 12-14% by 2050 compared to the 2000 production baseline (Nelson et al. 2009). Therefore, it will be impossible to meet the demand of increasing global population of almost 10 billion by 2050, unless revolutionary innovations similar to the green revolution of the 1960s or hybrid rice of the 1970s are forthcoming. These include technological interventions, reduction of production loss, expansion of rice cultivation in sub-optimal conditions (problem and saline soils) and the development of climate resilient varieties.

Gene mining and subsequent genetic manipulation are key technologies in abiotic stress tolerance research (Cabello et al. 2014; Mickelbart et al. 2015). However, although this reductionist approach has successfully characterized the function of a significant number of genes, the translation of these research outcomes into the field is scarce (Nelissen et al. 2014; Groen and Purugganan 2016; Gilliham et al. 2017) and the release of improved varieties is even rarer. The release and adoption of flash flood tolerant SUB1 rice varieties in several Asian countries are a notable example of the successful translation of research to fields/farmers although these varieties were developed through a marker-assisted backcrossing (MABC) strategy (Ismail et al. 2013). SUB1A, the master regulator of flash flood tolerance was identified from FR13A, a pure line selection from the local landrace, *Dhalputtia* grown in the Indian state of Orissa. The breakthrough point was the identification of a single QTL on chromosome 9 explaining nearly 70% of the phenotypic variation (Xu and Mackill 1996). Later, a specific gene was identified after concerted research efforts (Xu et al. 2006). Subsequently, multiple varieties were developed and released in several countries after field trials in numerous countries (Septiningsih et al. 2013). The success of SUB1 rice may be plausibly due to single gene regulated stress tolerance. Such a success story is unlikely to be repeated in other abiotic stress tolerances as dozens/hundreds of QTLs/genes have already been reported for their role in the respective stress tolerances (Cabello et al. 2014; Hu and Xiong 2014; Roy et al. 2014; Todaka et al. 2015; Ohama et al. 2017; Shakiba et al. 2017) and none of them can singly explain such high-level variations.

The stagnation of successful translation of abiotic stress tolerance research to fields/farmers is a growing concern (Nelissen et al. 2014; Groen and Purugganan 2016; Gilliham et al. 2017). This is emphasized by the impelling demand of climate resilient varieties because of the predicted impact of climate change on crop productivity together with the impending population demand. However, although current reductionist research has successfully identified and characterized significant numbers of abiotic stress tolerant genes (mostly in controlled environments), numerous factors of crop fields including macro/micro climate, seasonality, etc. are more highly diverse than can be simulated in controlled growth chambers or greenhouses. Therefore, increased stress tolerance of a transgenic plant in controlled growth chamber or greenhouse does not warrant the same performance in field conditions. In addition, almost all stress tolerance studies have simply overlooked the environmental aspect of stress tolerance, more specifically the eco-geographic aspect of stress-tolerant germplasms. However, environmental factors play an equally important role in the development of adaptive traits of plants. Simply put, whatever the genetic basis of the tolerance of a particular stress-tolerant local landrace, the tolerance was developed/acquired through recurrent exposure to the particular stress in a specific geo-climatic area and directional selection, either by farmers in domesticated crops or by nature. Put it in other words with a specific example; Why is *Dhalputtia*, the ultimate landrace from which SUB1A was identified, in Orissa, India; but not in Gansu, China or Sapporo, Japan? How important is the geolocation of tolerant accession? Is there any pattern of geolocation among abiotic stress tolerant rice accessions? An extraordinary genome-environment association study using the model plant *Arabidopsis thaliana* (Fournier-Level et al. 2011) clearly showed local adaption, and more specifically high fitness alleles were generally distributed closer to the site of specific and distinct climatic spaces. However, this kind of study has yet to be applied to rice accessions.

To examine our idea, we initially attempted a meta-analysis of geographic distribution patterns of abiotic stress-tolerant rice accessions/genotypes as reported earlier. However, we soon realized that the majority of the studies screened only a limited number of accessions/germplasms where specific geotagging information of those accessions/genotypes were not stated; mostly mentioning only the country of origin. Rice growing countries like India, China, Vietnam, or Myanmar etc. are latitude-wise very wide. Moreover, most of the rice growing countries have numerous agro-ecological and climatic zones. In addition, the majority of the screening studies did not follow the standardized evaluation protocol. However, different departments of International Rice Research Institute (IRRI) screened tens of thousands of rice accessions in the past for different abiotic stress tolerance. Moreover, they followed the standard evaluation system for rice (SES) (Table 1). In addition, geotagging information of these accessions are publicly available.

**Table 1:** Generalized Standard Evaluation System for rice (SES)

SES score^a^	General Description^b^ and Desirability	Cold Stress at Seedling Stage^c^	Tolerance^a^
0	Absence of visible symptoms or injury	no damage to leaves, normal leaf color	Highly tolerant
1	Potential donor of the trait		
2	Trait expression is satisfactory	tip of leaves slightly dried, folded, and light green	Tolerant
3	Potential donor of the trait		
4	Trait expression is not as good as it should be, but may be acceptable under some circumstances	intermediate	Intermediate
5			
6		leaves of seedlings are severely rolled and dried	Sensitive
7	Trait expression is unsatisfactory (not useful)		
8		the majority of the seedlings died and dried	Highly sensitive
9			

^a^Although SES scoring is a 10 division (0-9) visual or pictorial scoring system; however, for some traits, human eye cannot easily differentiate between 10 divisions, then only five (1, 3, 5, 7, 9) or three (1, 5, 9) digits are used (IRRI 2013). In this review, generally we considered SES 1-3, 4-6, 7-9 as tolerant, intermediate, sensitive, respectively

^b^Specific description depends on the stress, severity, growth stage of the plants etc.

^c^Most rice scientists, especially rice breeders are familiar with SES scoring, however, those unfamiliar can simply understand the interpretation of generalized SES scoring system with the specific SES scoring examples of cold stress at seedling stage

Generally, abiotic stresses are studied, analysed, and even reviewed separately; rarely multiple stresses are analysed together. However, in this review, we have critically analysed the geographic distribution patterns of highly stress tolerant rice accessions for all major abiotic stresses along with one micronutrient deficiency. If the geographic distribution pattern of abiotic stress tolerant accessions showed a shared pattern, then the genetic basis of local adaptation or high fitness alleles can be identified through further studies utilizing the advances in genome research tools and know-hows such as genome sequencing and genome wide association studies (GWAS). Remarkably, the cost of genome sequencing has been drastically reduced in the last two decades due to the revolutionary advancement of DNA sequencing technologies. Therefore, sequencing of thousand accessions is no longer a dream project and has already become a reality (Alexandrov et al. 2014). Thus, we have now both the toolbox as well as the know-how to identify the genetic signature from big-data, we just need an effective approach to overcome the limitations.

Therefore, in this review, we have explicitly examined the results of large-scale screening of abiotic stress tolerance to identify the preferential geographic distribution patterns of all major abiotic stress-tolerant rice accessions. Abiotic stresses include cold, salt, alkali, drought, and both flash and prolonged floods. Alkaline (sodic) soils are usually zinc deficient, therefore, we have also analysed zinc deficiency tolerant accessions to confirm whether salt, alkali and zinc deficiency tolerant accessions show a shared pattern.

## Review

### Geographic Distribution Pattern of Abiotic Stress Tolerant Rice Accessions

#### Salt Tolerance

**Fig. 1:**
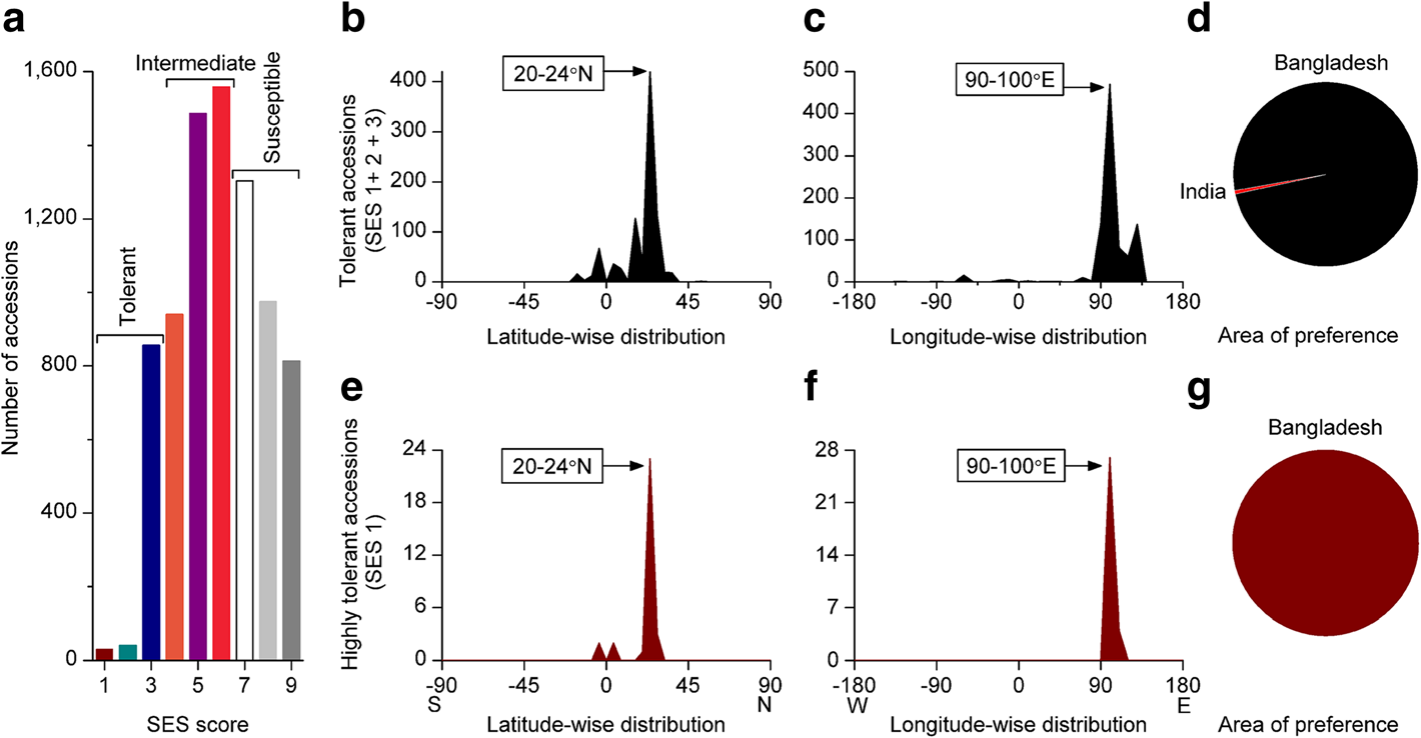
Geographic distribution pattern of salt tolerant rice accessions. **a** Extent of salt tolerant accessions, **b**-**d** Geographic distribution pattern of salt tolerant (SES score 1-3) accessions, **b** Latitude-wise distribution, **c** Longitude-wide distribution, **d** Area of preference, **e**-**g** Geographic distribution pattern of highly salt tolerant (SES score 1) accessions, **e** Latitude-wise distribution, **f** Longitude-wide distribution, **g** Area of preference. Screening results of all abiotic stress tolerance were collected from the International Rice Genebank Collection Information System (IRGCIS 2017). Geo-tagging information of the tolerant and highly tolerant accessions were plotted for latitude- and longitude-wise distribution of 4° and 10° band, respectively. The area of preference was identified based on the prevalence of tolerant or highly tolerant accessions within the preferred latitude and longitude. List of highly tolerant accessions can be found in Additional file 4: Table S1

We first analysed the extent of salt tolerance accessions from a very large-scale screening comprised of more than 8000 accessions, originating from 39 countries or territories. The majority of accessions were moderate to salt susceptible whereby nearly 12% accessions were tolerant (SES score 1-3) (Fig. 1a). However, highly salt tolerant accessions were very rare, e.g. only 0.39% of accessions. We analysed the geographic distribution pattern of only salt tolerant accessions having a SES score of 1-3 (Fig. 1b-g) and identified preferred latitude (4° band), longitude (10° band) and area of preference of tolerant accessions. The area of preference was identified based on the prevalence of tolerant accessions within the preferred latitude and longitude band. Interestingly, both latitude- and longitude-wise distribution of tolerant and highly tolerant accessions clearly showed single peak preferences (Fig. 1b-c, e-f), and both were completely within the same latitude (20-24°N) and longitude band (90-100°E). Remarkably, within the preferred latitude and longitude band, almost all tolerant (Fig. 1d) and all highly tolerant accessions (Fig. 1g) originated from Bangladesh.

To reconfirm the area of preference of salt tolerant rice accessions, we analysed the results of a recent medium-scale screening (Platten et al. 2013) that comprised hundreds of accessions of both of the cultivated rice, *Oryza sativa* and *O. glaberrima*. However, although only seven accessions of *O. sativa* were found highly tolerant in that study, highly tolerant accessions were also prevalent in Bangladesh (Bangladesh 4, India 1, Philippines 1, and Thailand 1). We analysed the geographic distribution pattern of the 50 salt tolerant accessions (7 and 43 accessions of highly tolerant and tolerant category, respectively). Interestingly, the preferred latitude, longitude, and the area of preference of salt tolerant accessions remained the same (Additional file 1: Figure S1a-c, identical to the large-scale screening, Fig. 1b-g). Thus, the recurrence of Bangladesh as the area of preference of highly salt tolerant rice accessions in independent studies clearly validated the specific distribution pattern of salt tolerant rice.

#### Alkaline Tolerance

**Fig. 2:**
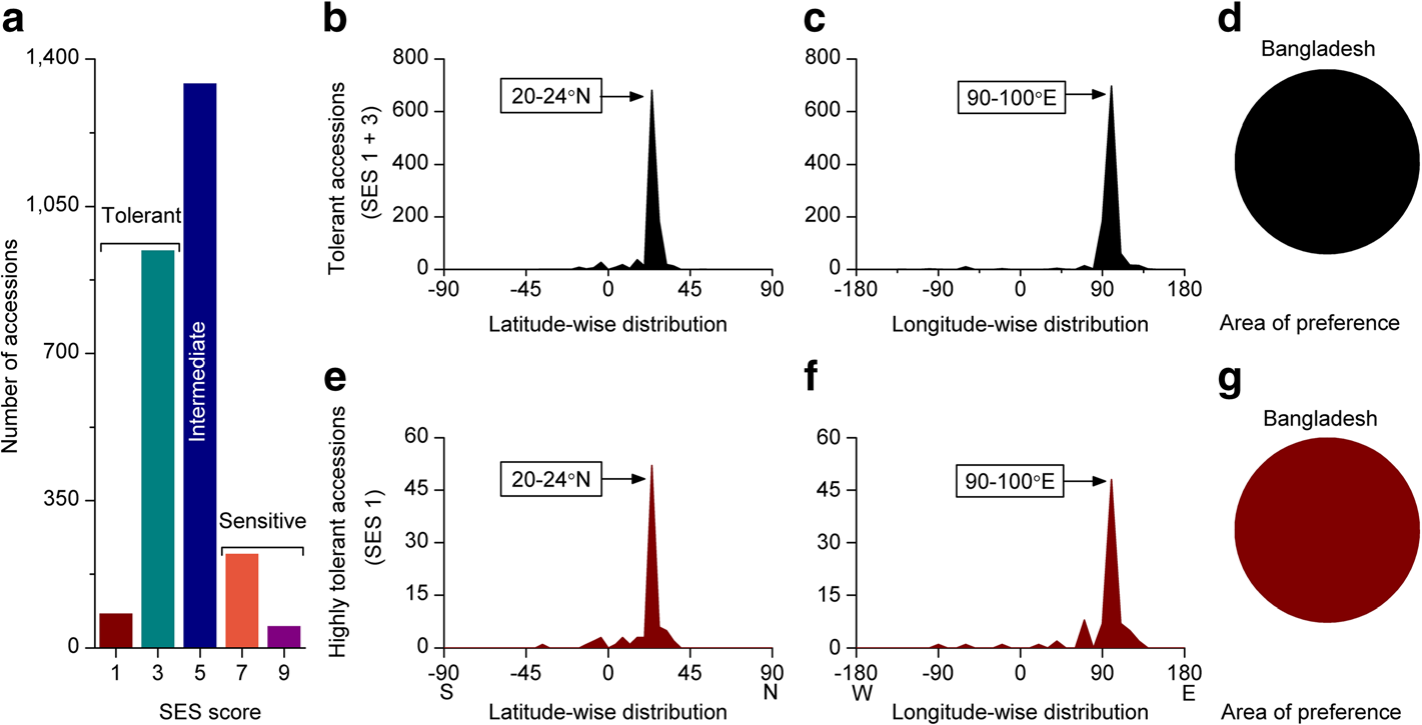
Geographic distribution pattern of alkali tolerant rice accessions. **a** Extent of alkali tolerant accessions, **b**-**d** Geographic distribution pattern of alkali tolerant (SES score 1-3) accessions, **b** Latitude-wise distribution, **c** Longitude-wide distribution, **d** Area of preference, **e**-**g** Geographic distribution pattern of highly alkali tolerant (SES score 1) accessions, **e** Latitude-wise distribution, **f** Longitude-wide distribution, **g** Area of preference. List of highly tolerant accessions can be found in Additional file 4: Table S1

Hundreds of million hectares of Asia, Pacific and Australia (over 50% salinity affected areas) are categorized as alkaline (sodic) soils having a higher pH, usually greater than 8.5, sometimes over 10. Therefore, we identified the area of preference for alkali tolerance and checked whether the preferred latitude, longitude, and the area of preference was in accordance with that of the salinity tolerance. The extent of alkali tolerance clearly showed that tolerant accessions were relatively common, e.g., nearly 40% of screened accessions (Fig. 2a). However, only 3.13% accessions were highly tolerant. Remarkably, the geographic distribution patterns of both tolerant and highly tolerant accessions were exactly the same (Fig. 2b-g) with regard to salt tolerance. Likewise, within the preferred latitude and longitude band, all tolerant and highly tolerant accessions originated from Bangladesh (Fig. 2d, g).

#### Zinc Deficiency Tolerance

**Fig. 3:**
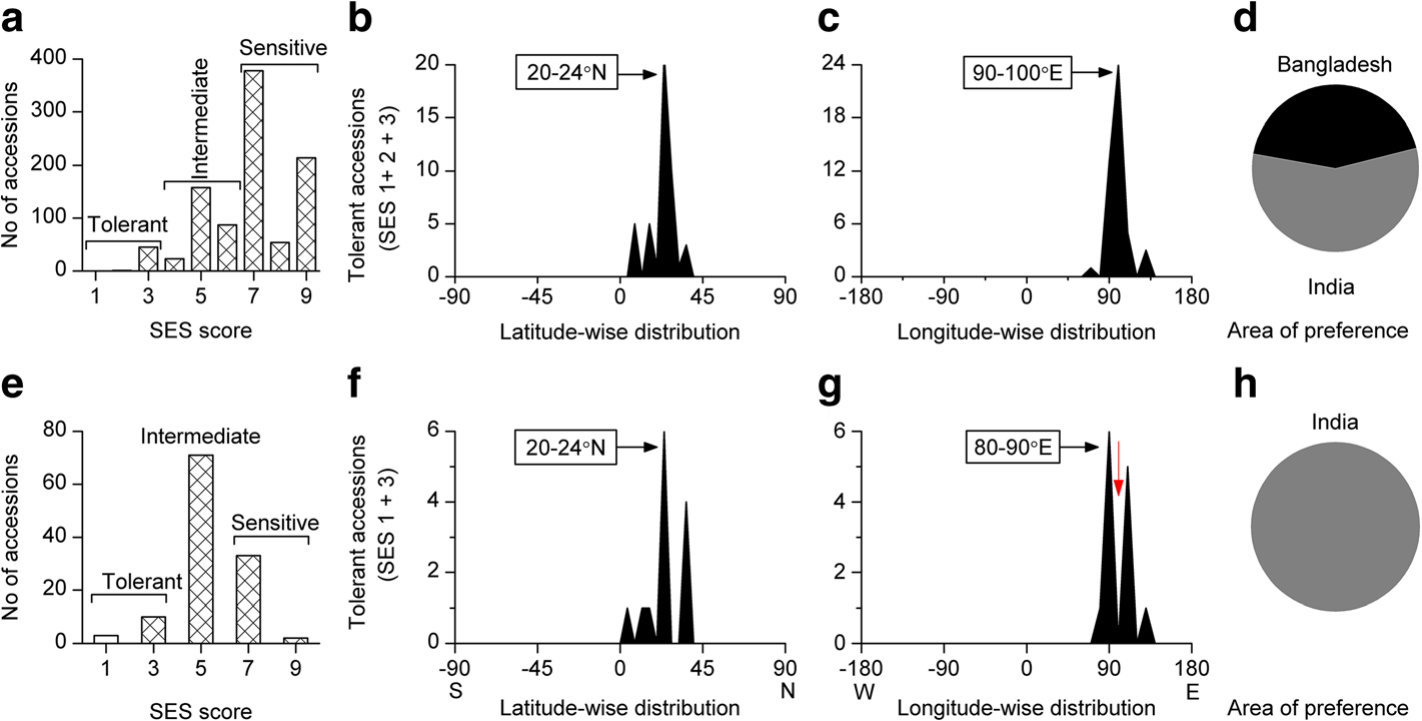
Geographic distribution pattern of zinc deficiency tolerant rice accessions. **a**-**d** Zinc-deficient field screening, **a** Extent of tolerant accessions **b** Latitude-wise distribution, **c** Longitude-wide distribution, **d** Area of preference, **e**-**h** Greenhouse screening, **e** Extent of tolerant accessions, **f** Latitude-wise distribution, **f** Longitude-wide distribution, **g** Area of preference. List of highly tolerant accessions can be found in Additional file 4: Table S1

We used another approach to verify the area of preference of salinity and related stress tolerances. Alkaline soils are rich in free carbonate and bicarbonate along with excess sodium, therefore, alkaline soils are usually zinc deficient (Forno et al. 1975; Alloway 2008). Thus, the area of preference of zinc deficiency tolerant accessions should be logically in the same area of preference of alkali tolerance. We analysed zinc deficiency tolerant accessions from zinc-deficient field (Fig. 3a-d) and greenhouse (Fig. 3e-h) screenings. Only 4.8% (Fig. 3a) and 10.9% (Fig. 3e) accessions were found zinc deficiency tolerant in the field and greenhouse screenings, respectively. However, although 3 accessions showed high tolerance in greenhouse screening (Fig. 3e), not a single accession was found to be highly tolerant in the field screening (Fig. 3a). Remarkably, the preferred latitude of zinc deficiency tolerant accessions (20-24°N) also showed the identical preferred latitude of salt and alkali tolerances. Similarly, the preferred longitude of tolerant accessions of zinc-deficient field screening (Fig. 3a-d) also showed similar patterns (90-100°E) of salt and alkali tolerance. However, that of greenhouse screening was not identical to field screening, which was rather slightly shifted to an adjacent longitude band, i.e., 90-100°E to 80-90°E (Fig. 3 c, g). To confirm why the preferred longitude band slightly shifted between the screenings, we analysed the geolocation/country of origin of all accessions used in the greenhouse screening. Remarkably, we found that not a single accession originating from Bangladesh was screened in the greenhouse screening. Therefore, exclusion of rice accessions originated from Bangladesh in the greenhouse screening certainly explains the shifting of preferred longitude band of zinc-deficient field (Fig. 3a-d) and greenhouse (Fig. 3e-h). However, although India turned out to be marginally the area of preference for zinc deficiency tolerant accessions in zinc-deficient field screenings (Fig. 3d), a detailed analysis of the geolocation of the tolerant accessions revealed that the majority of the zinc deficiency tolerant Indian accessions (IRGC 12310, 12314, 12337, 12370, 12567, 12592, 12612, 12616) had originated from areas very close (20–40 km) to the Bangladesh border. Therefore, we considered Bangladesh the area of preference of zinc deficiency tolerance too.

#### Prolonged Flood Tolerance

**Fig. 4:**
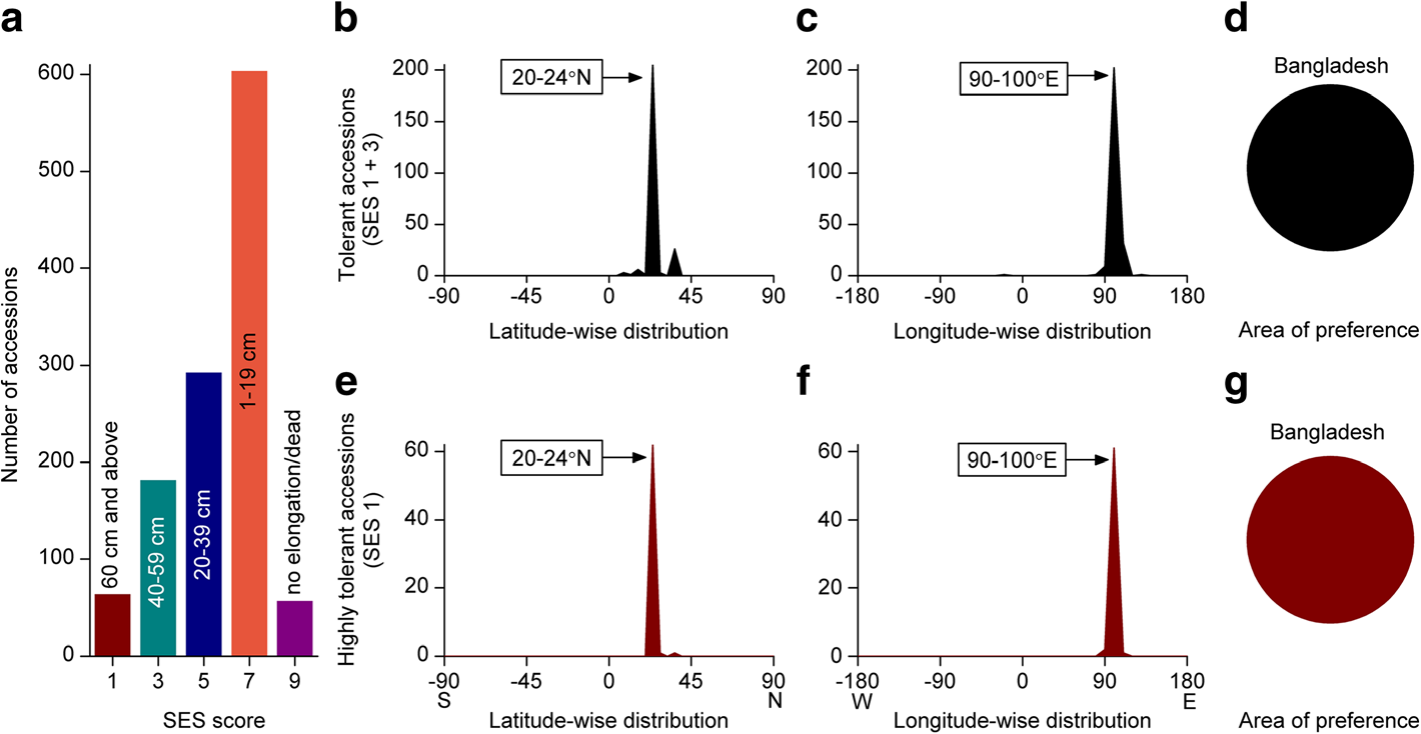
Geographic distribution pattern of highly elongating (prolong flood tolerant) rice accessions. **a** Extent of elongation of rice accessions in 1-m water depth, **b**-**d** Geographic distribution pattern of 40 cm or above elongating (SES score 1-3) accessions, **b** Latitude-wise distribution, **c** Longitude-wide distribution, **d** Area of preference, **e**-**g** Geographic distribution pattern of highly elongating (60 cm and above, i.e., SES 1) rice accessions, **e** Latitude-wise distribution, **f** Longitude-wide distribution, **g** Area of preference. List of highly elongating accessions can be found in Additional file 4: Table S1

Both cultivated rice species (*Oryza sativa* and *O. glaberrima*) have deepwater ecotypes that possess the special capacity of internode elongation after rising flood water. Therefore, deepwater rice ecotypes are usually prolonged flood (1-5 months) tolerant. We identified the area of preference of deepwater or prolonged flood tolerant rice by two different approaches. Initially, we analysed the preferential distribution pattern of highly elongating rice accessions from the screening results of elongation rates of 6-week-old rice plants. Highly elongating accessions (5.3%) were the highest among all analysed abiotic stresses (Fig. 4a). Remarkably, preferred latitude, longitude, and the area of preference of highly elongating rice accessions showed exactly the same pattern (Fig. 4b-g) as for other abiotic stresses.

**Fig. 5:**
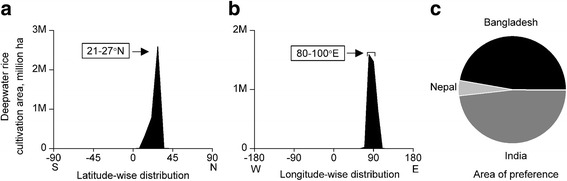
Geographic distribution pattern of deepwater rice cultivation area. **a** Latitude-wise distribution, **b** Longitude-wide distribution, **c** Area of preference

Finally, we analysed latitude- and longitude-wise distribution patterns of deepwater rice cultivation areas to identify the preferred area of deepwater rice cultivation as well as to verify the area of preference of highly elongating accessions in accordance with the preferred area of deepwater rice cultivation. Nearly 4-million-hectare areas were classified as deepwater rice growing areas (Huke and Huke 1997). However, deepwater rice cultivation areas were reported at mostly state or province levels for the majority of the deepwater rice growing countries (Huke and Huke 1997), where numerous states/provinces are located over 4° latitudes, therefore, we analysed a 7° latitude band instead of 4° to reduce the boundary concerns. Interestingly, almost 70% deepwater rice areas belong to Bangladesh, India, and Nepal within the preferred latitude (21-28°N) and longitude (80-100°E) (Fig. 5a-c). India accounted for marginally more deepwater rice cultivation areas (2%) than Bangladesh within the preferred latitude and longitude. However, the majority of the deepwater rice cultivation areas of India belong to Bangladesh- adjacent Indian states in both eastern and western borders. Therefore, we considered Bangladesh centred area as the area of preference of deepwater rice.

#### Flash Flood Tolerance

**Fig. 6:**
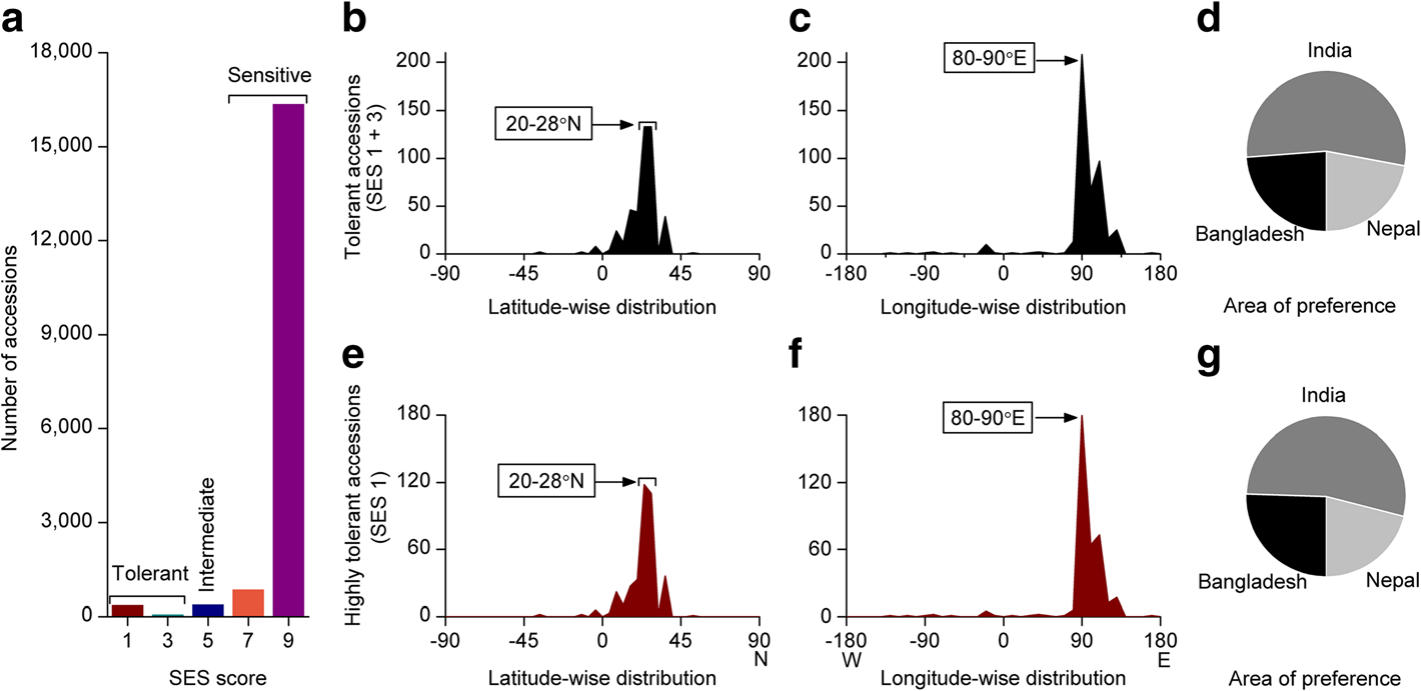
Geographic distribution pattern of flash flood tolerant rice accessions. **a** Extent of flash flood tolerant accessions, **b**-**d** Geographic distribution pattern of flash flood tolerant (SES score 1-3) accessions, **b** Latitude-wise distribution, **c** Longitude-wide distribution, **d** Area of preference, **e**-**g** Geographic distribution pattern of highly tolerant (SES score 1) accessions, **e** Latitude-wise distribution, **f** Longitude-wide distribution, **g** Area of preference. List of highly tolerant accessions can be found in Additional file 4: Table S1

Sudden heavy rainfall may cause flash flooding in low-lying rice growing areas. Over 18,000 accessions were analysed for flash flood tolerance. The majority of the screened accessions (95%) were highly sensitive. Only 2.1% accessions were highly flood tolerant. The majority of the highly flash flood tolerant accessions originated from three adjacent countries: Bangladesh, India, and Nepal within the preferred latitude (20-28°N) and longitude (80-90°E) (Fig. 6a-g). Indian accessions accounted for more than half of the tolerant accessions (Fig. 6d, g), however, Indian accessions mostly originated from Bangladesh and Nepal adjacent Indian states. Remarkably, total number of Indian accessions (3734) screened for flood tolerance was nearly double than that of Bangladesh (2175). A recent study of submergence tolerant rice accessions of Bangladesh showed that the survival percentage of numerous Bangladeshi accessions, like Saita (79.1%), Damsi (83%) Kalojoma (83.3%), Putidepa (83.7%), Lakhi (89.5%) were similar to that of FR13A (85.4%) or even higher in DG1-349 (93.4%), DSL-78-8 (95.4%), after 16 days submergence (Iftekharuddaula et al. 2016). However, some submergence tolerant accessions, like DG1-349, Kalojoma, DSL-78-8 do not possess the same resistance allele as FR13A (Iftekharuddaula et al. 2016). Thus, these accessions were considered as potential genetic donors for identifying novel submergence-tolerance QTLs. Earlier Pucciariello and Perata (2013) hypothesized that submergence tolerance may have been introgressed into domesticated rice from wild rice whereby the introgression probably occurred in the Ganges Basin. Taking all these facts into consideration, we conclude that the Bangladesh centred area is the area of preference of flash flood tolerant rice accessions too.

#### Anaerobic Germination (AG) Tolerance

**Fig. 7:**
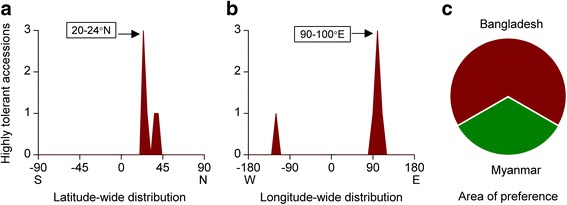
Geographic distribution pattern of anaerobic germination (AG) tolerant rice accessions. **a** Latitude-wise distribution, **b** Longitude-wide distribution, **c** Area of preference

Sudden flooding due to heavy rainfall or even waterlogging can limit seedling establishment in direct seeded rice fields since rice is sensitive to submerged seed germination. Flash flood tolerance during seed germination, i.e., anaerobic germination (AG) tolerance is the rarest among all abiotic stress tolerances. Over 8000 accessions and breeding lines have previously been analysed for AG tolerance and an initial screening identified only 19 accessions with over 70% survival (Angaji et al. 2010). Repeated experiments reduced highly tolerant accession to 6, i.e., only 0.07% accessions were highly tolerant (Ismail et al. 2009). Among the 6 highly tolerant accessions, Khaiyan, Kalongchi originated from Bangladesh whereas Nanhi, Khao Hlan On, Ma-Zhan Red and Cody are from India, Myanmar, China and USA, respectively. Surprisingly, despite the very limited number of highly tolerant accessions, the preferred latitude (20-24°N), longitude (90-100°E) and the area of preference (Bangladesh) were still the same (Fig. 7a-c) as other stress tolerances.

#### Drought Tolerance

**Fig. 8:**
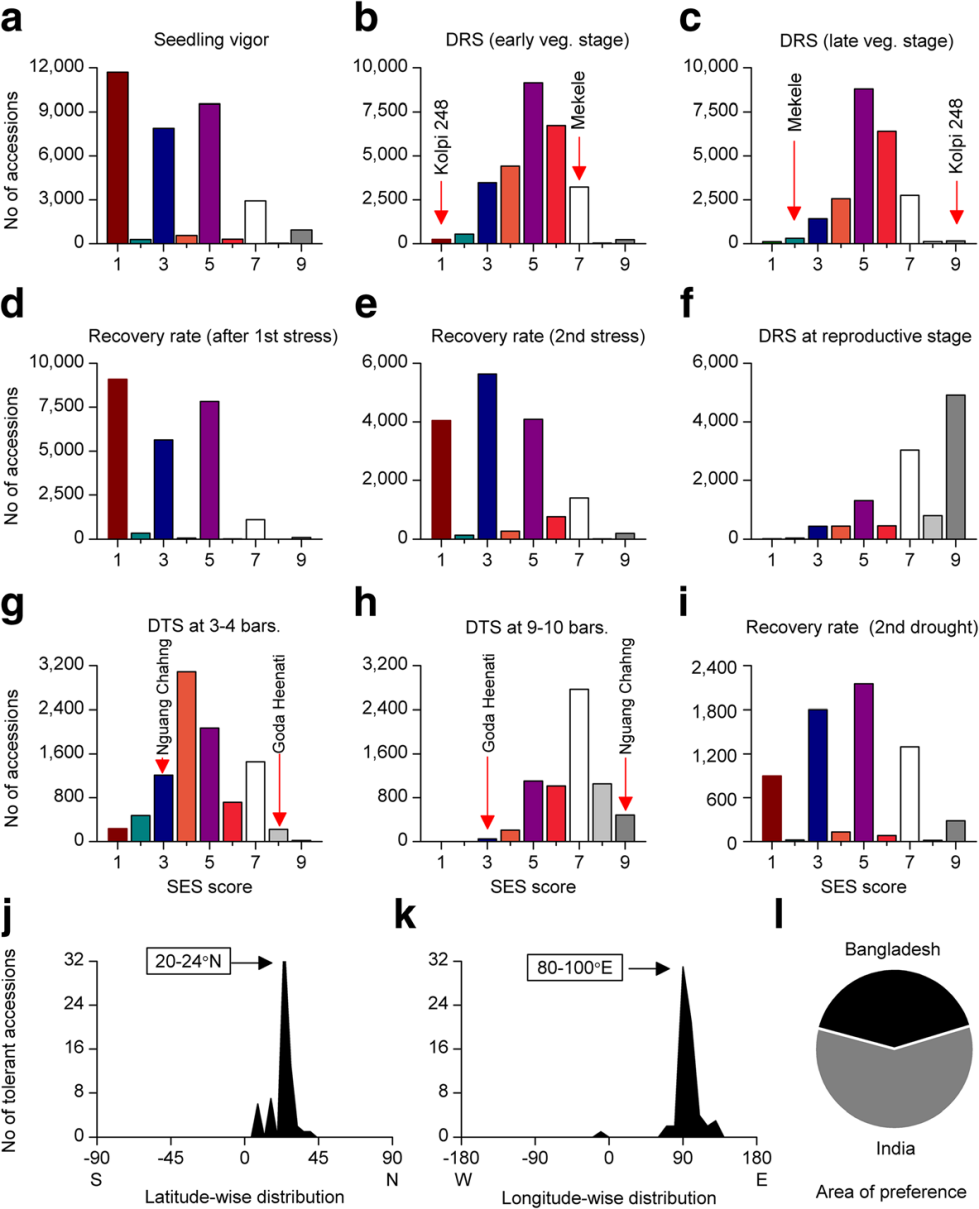
Extent of tolerant accessions in different drought and related screening experiments. **a** Seedling vigour scoring taken 15 days after sowing in upland culture, **b** Drought resistance score (DSR) at early vegetative (tillering) stage (50 to 60 days) of both the early and late-maturing varieties, **c** Drought resistance score at late vegetative stage (80-100 days) of late-maturing varieties, **d** Rate of recovery after the 1st stress, **e** Rate of recovery after the 2nd stress, **f** Drought resistance score at reproductive stage, **g** Drought tolerance score of accessions at 3-4 bars soil moisture, **h** Drought tolerance score of accessions at 9-10 bars soil moisture, **i** Rate of recovery after exposure to 2nd drought. Scores were taken after 10 days following soaking rain or watering **j**-**k** Geographic distribution pattern of drought tolerant accessions identified by direct selection, i.e., yield performance in field conditions, **j** Latitude-wise distribution, **k** Longitude-wide distribution, **l** Area of preference. List of tolerant accessions can be found in Additional file 4: Table S1

Drought is generally considered the most complex and the toughest stress to overcome. Therefore, the highest number of attempts (from seedling vigour to drought recovery) and accessions (total 159,812 entries from 38,433 different rice accessions) were screened to identify highly drought tolerant rice accessions (Fig. 8a-i). However, drought tolerance is often growth stage and environment specific. For instance, 11,700 (34%) (Fig. 8a), 9097 (38%) (Fig. 8d) and 4046 (24%) (Fig. 8e) accessions were found highly tolerant in the respective screening of seedling vigor, rate of recovery after the first and second stress, respectively. Against this, only 248 (0.88%) (Fig. 8b) and 110 (0.49%) (Fig. 8c) accessions were found to be highly tolerant in early or late vegetative stages drought screening. However, not a single accession was found having SES 1 after severe drought condition (Fig. 8h). More interestingly, some rice accessions such as Kolpi 248 (IRGC 3725) or Early 3 (IRGC 38708) were found to be highly tolerant (SES 1) in early vegetative stage drought screening while the same accessions were highly sensitive (SES 9) after late vegetative stage drought exposure (Fig. 8b-c). In opposition to this, accessions such as Meleke were highly tolerant (SES 2) at late vegetative stage drought while the same accession was sensitive (SES 7) after early vegetative stage drought. Similar patterns were also observed in the mild or severe drought conditions. For instance, Nguang Chahng (IRGC 64558) was drought tolerant after mild drought stress (bars 3-4) while it was highly sensitive (SES 9) after severe drought (9-10bars). In contrast, Goda Heenati (IRGC 15419) was found to be tolerant after severe drought whereas it did not perform well (SES 8) even after mild drought stress (Fig. 8g, h). To identify why drought tolerance or SES score of these accessions varied so much, we compared their SES scoring patterns with Dular (IRGC 10615), one of the most well-known and highly drought tolerant rice accession. The SES scores of Dular in the early, late vegetative and reproductive stage drought screening was SES 4, 2, and 1, respectively, i.e., varied slightly. Therefore, the contrasting SES scores/tolerance (SES 1-3 and 7-9) of the same accession (such as Kolpi 248) in different growth stages clearly suggest the drought tolerance of these accessions are possibly growth stage dependent. It is noteworthy to mention that flowering and heading stages are the most sensitive stages to water deficit in rice. Therefore, rice yield is severely reduced after drought exposure in the reproductive stage. Remarkably, the majority of the reproductive stage drought tolerant accessions originated from Bangladesh (129) and India (112).

Between 1978 and 1985, nearly 40,000 germplasms (accessions and breeding lines) were screened for the best drought tolerant germplasm; however, the majority of the top 20 outstanding germplasms were breeding lines (de Datta et al. 1988). The sources of tolerance in breeding lines are often obscure as the breeding process involves numerous parents. For instance, the ancestry of the most popular rice variety, IR64 includes 19 landraces from nine different countries (Khush and Virk 2005). All these large-scale drought and related screening results clearly showed that drought tolerance largely depends on growth stage, severity of drought and the specific environmental conditions. Most importantly, drought tolerance for vegetative stages does not correlate with yield performance under water stress. Moreover, drought stress breeding is challenging due to the lack of suitable screening methods.

A recent selection approach, i.e., direct selection for yield performance in both drought and well-watered conditions seems a most effective selection strategy and has been increasingly accepted for drought tolerance studies in rice (Kumar et al. 2008; Venuprasad et al. 2008; Torres et al. 2013). Using this strategy, 988 accessions originated from 47 countries were screened in fields for yield performance under drought and well-watered conditions during 2004-2009 (Torres et al. 2013). We analyzed the geographic distribution pattern of the tolerant accessions (Fig. 8 j-k) identified in that study. Remarkably, both latitude- and longitude-wise distribution patterns of the drought tolerant accessions also clearly showed a single peak preference where the preferred latitude (20-24°N) and longitude (80-100°E) were identical to other abiotic stress tolerances (Fig. 8j-i). The preferred area of drought tolerance within the preferred latitude and longitude comprised more than half of the tolerant accessions (74% considering the entire countries). India accounted for marginally more tolerant accessions than Bangladesh (Fig. 8l); however, the majority of the Indian accessions originated from Bangladesh adjacent states. Moreover, recommended varieties for drought stress breeding such as Kataktara Da2, Dular, Shada Shaita, and DA 28 (Torres et al. 2013) also originated from Bangladesh and most of them are still being cultivated in considerable areas in Bangladesh (Hossain et al. 2013). Therefore, based on the consistent prevalence of drought tolerant accessions in Bangladesh centred area in both reproductive stage drought screening as well as direct selection by yield performance, we considered Bangladesh centred area also the area of preference of drought tolerant rice accessions.

#### Cold Tolerance

**Fig. 9:**
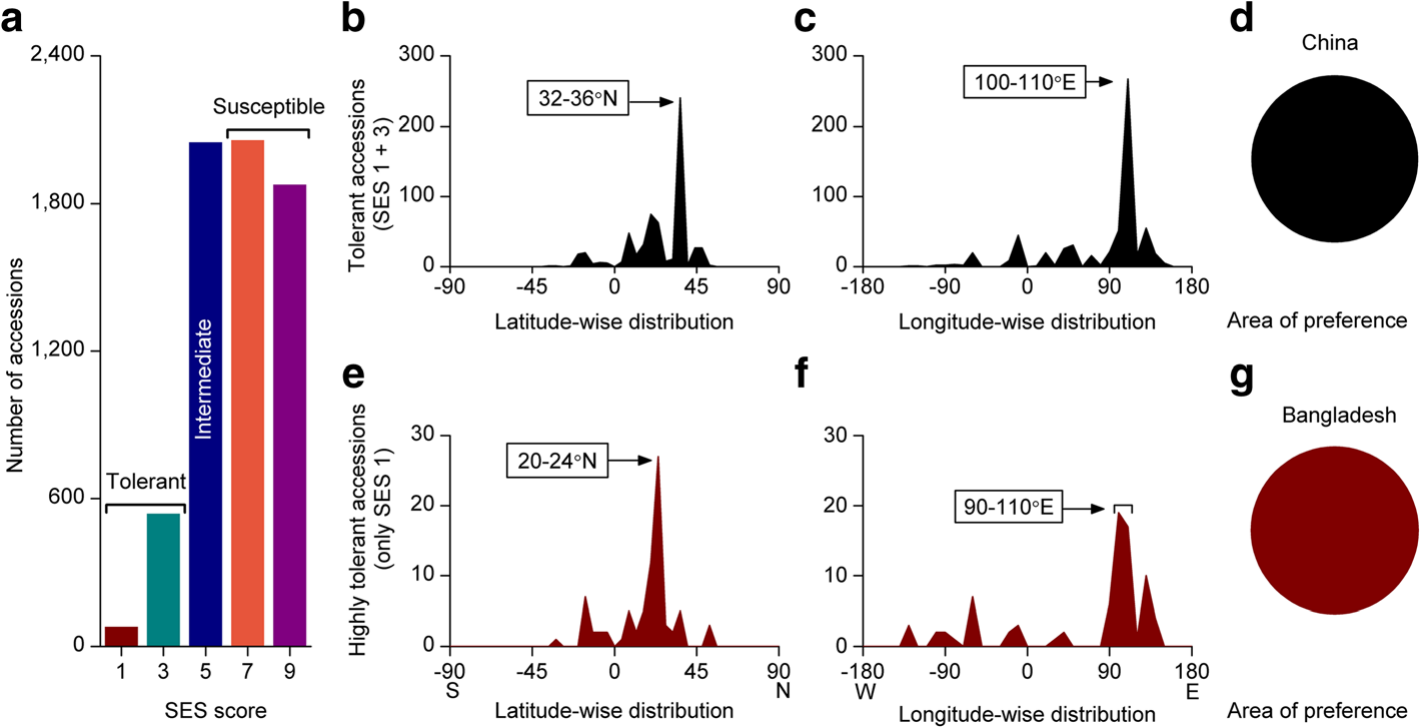
Geographic distribution pattern of cold tolerant rice accessions. **a** Extent of cold tolerant accessions, **b**-**d** Geographic distribution pattern of cold tolerant (SES score 1-3) accessions, **b** Latitude-wise distribution, **c** Longitude-wide distribution, **d** Area of preference, **e**-**g** Geographic distribution pattern of highly cold tolerant (SES score 1) accessions, **e** Latitude-wise distribution, **f** Longitude-wide distribution, **g** Area of preference. List of highly tolerant accessions can be found in Additional file 4: Table S1

We finally analysed the extent of cold stress tolerant accessions from a very large-scale screening that comprised more than 6000 rice accessions originating from 77 countries or territories. The majority of the screened accessions were moderate to cold susceptible where only below 10% accessions were cold tolerant (Fig. 9a). Both latitude- and longitude-wise distributions of the cold tolerant accessions (SES score 1-3) showed a single peak preference (Fig. 9b, c), despite the occurrence of tolerant accessions in the wider spectrum. All the tolerant accessions within the preferred latitude (32-36°N) and longitude (100-110°E) originated from Gansu, the north-central province of China (Fig. 9d). Taking an SES score of 1 as highly tolerant, only 1.22% of accessions were highly cold tolerant (Fig. 9a). However, the geographic distribution pattern of these highly tolerant accessions showed a completely different preferred latitude (20-24°N) and longitude (90-110°E) (Fig. 9e, f) than that of the tolerant category. Remarkably, all highly tolerant accessions within the preferred latitude and longitude originated from Bangladesh (Fig. 9g).

**Fig. 10:**
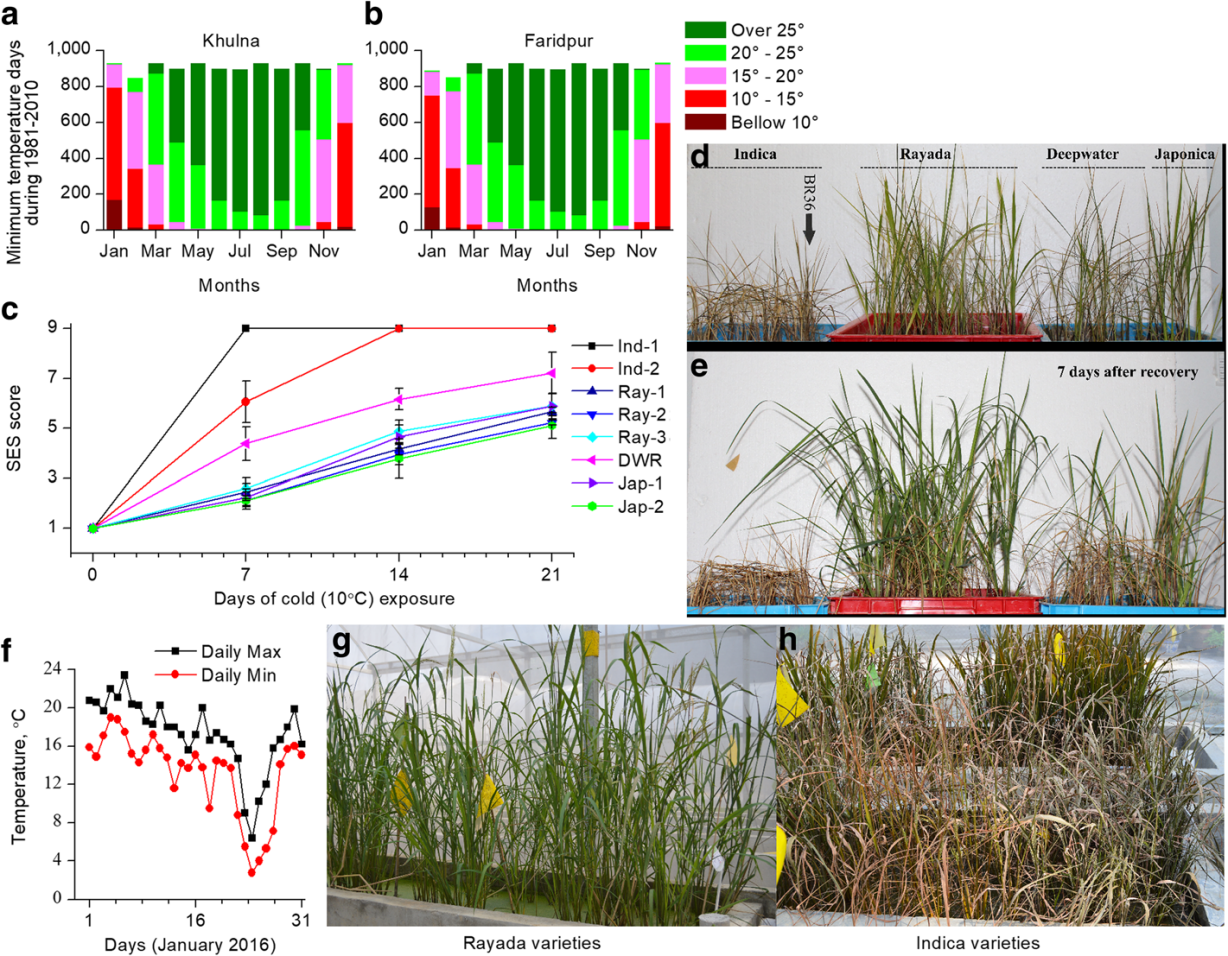
Extent of cold tolerance of Rayada rice, a Bangladesh confined deepwater rice ecotype. **a**-**b** Minimum temperature days at the two weather stations, neighboring Rayada rice growing areas, **a** Khulna, **b** Faridpur, Bangladesh, **c**-**e** Comparative cold tolerance of different rice varieties conducted in growth chamber, **c** SES score, **d** Representative phenotypes after prolong cold exposure (continuous 21 days of 10 °C), **e** Representative phenotypes 7 days after recovery at 28 °C, **f**-**h** Comparative cold tolerance at field conditions, **f** Temperature profile at the experimental site during January 2016, collected from the nearest automated weather station. **g** Representative phenotypes of Rayada varieties 7 days after the cold-wave, **h** that of the Indica varieties. Ind 1, 2 and Jap 1, 2 represent BR 11, BRRI 36, Donjgin, Nipponbare, respectively where BRRI 36 is cold tolerant indica variety. Different rice varieties used in the extent cold stress tolerance were collected from International Rice Research Institute, Bangladesh Rice Research Institute, National Institute of Agrobiological Sciences, Japan and Pohang University of Science and Technology, Korea

Normally, the japonica type, especially temperate japonica type rice varieties are more cold tolerant than indica varieties. Bizarrely, coincidence of the area of preference of highly cold tolerance rice accessions in Bangladesh along with all other tolerant abiotic stresses made us curious to examine the long-term minimum temperature days of Bangladesh, especially in Rayada rice growing areas. Rayada rice is the most primitive deepwater ecotype (Bin Rahman and Zhang 2013) that still shares some features of wild rice (Khush 1997). Moreover, Rayada rice is completely confined to Bangladesh. Furthermore, it has almost a year-long life cycle. Thus, Rayada rice faces all sort of annual stresses including cold (Bin Rahman and Zhang 2013). We therefore analyzed the number of minimum temperature days over a 30 year period (1980-2010) at two weather stations (Khulna and Faridpur, Bangladesh) neighboring the Rayada rice growing areas (Fig. 10 a, b). It clearly showed November to February is the winter season, whereby January is the coldest month, usually below 15 °C, occasionally even below 10 °C. Since Rayada varieties are sown in Nov-Dec, Rayada seedlings are naturally exposed to a cold stress of below 15 °C or even below 10 °C.

To compare and experimentally verify the cold tolerance capacity of Rayada rice, we imposed a cold stress of 10 °C for a continuous 21 days on 35 day old seedlings in a growth chamber. Interestingly, all of the indica rice varieties we screened, including high yielding cold tolerant indica variety, BRRI 36 completely died and were dessicated in the prolong cold exposure (Fig. 10c-e). In contrast, Rayada rice accessions showed a similar cold tolerance ability to the two temperate japonica varieties, Nipponbare and Dongjin. Remarkably, the recovery performance of Rayada varieties was even better than that of the japonica varieties (Fig. 10e). To reconfirm the cold tolerance of mature Rayada plants under field conditions, we grew different rice varieties including Rayada rice in the CUHK gene garden in three consecutive winter seasons from 2014 to 2016. However, winter temperature of 2014, 2015 did not drop below 10 °C on consecutive days. Fortunately, between 22 and 27 January of 2016, the temperature of Hong Kong dropped below 10 °C for 5 consecutive days (Fig. 10 f). Remarkably, the temperature of 24th January 2016 was 3.1 °C which is the lowest recorded temperature for over 50 years in Hong Kong. Exposure to these chilling temperatures on Rayada varieties showed no symptoms (Fig. 10 g) while leaves of indica varieties became severely rolled and dried (Fig. 10 h). Thus, cold tolerance ability of Rayada rice in both seedling and mature plants were experimentally confirmed.

### Agro-climatic Features of the Area of Preference for All Abiotic Stress Tolerance Rice

**Fig. 11:**
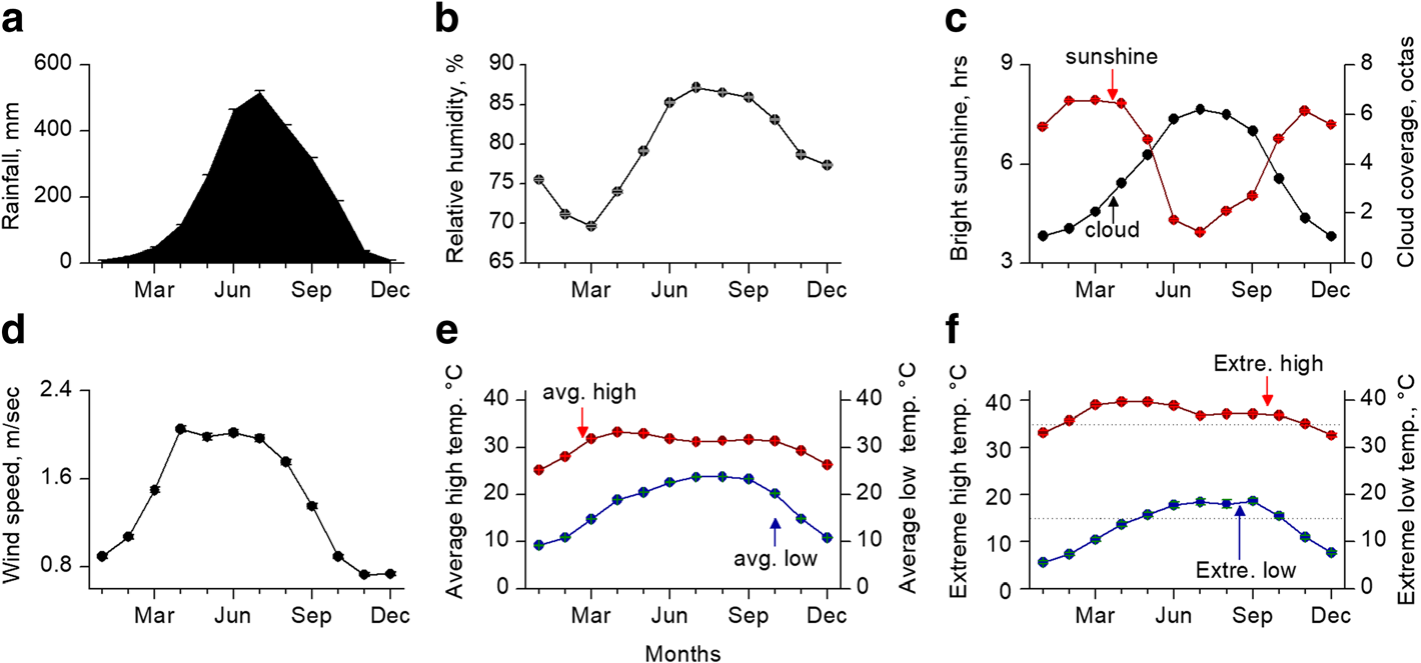
Climatic features of Bangladesh. **a** Rainfall, **b** Relative humidity, **c** Bright sunshine hours and cloud coverage, **d** Wind speed, **e** Average high and low temperature, **f** Extreme high and low temperature (dotted lines represent the thresholds of rice cultivation). Climatic data were collected from Bangladesh Meteorological Department and climate information management system of Bangladesh Agricultural Research Council

**Fig. 12:**
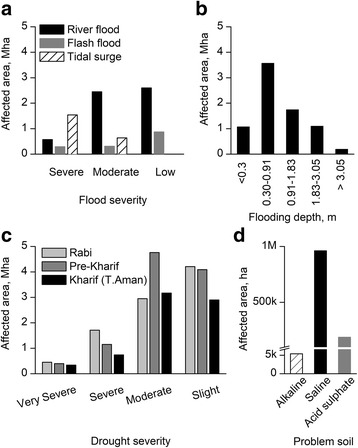
Extent of the agroecological constrained areas of Bangladesh **a** Flood-prone areas by flooding types. Severity of flooding is classified based on the recurrence of flooding and the extent of crop damage. Severe represents occurrence of floods 5 times or more in 10 years timeframe with considerable loss/damage of standing crops, where moderate means 3-4 times occurrence with significant loss/damage while low represents occurrence at least twice in 10 years timeframe with loss of standing crops. **b** Flood-prone areas by flooding depth, **c** Drought-prone areas by crop seasons. Severity of drought is classified based on the extent of yield reduction. Very severe, severe, moderate, and slight severity represent yield reduction of more than 50%, 30-50%, 15-30% and below 15%, respectively, **d** Other agroecological constrained areas. (Map of the agroecologically constrained areas of Bangladesh is shown in Additional file 3: Figure S3). Information of Agro-ecological constrains areas of Bangladesh were collected from land resources information management system of Bangladesh Agricultural Research Council

Since all abiotic stress tolerant rice accessions predominately originated from Bangladesh or Bangladesh centred areas, we therefore decided to examine both climatic (Fig. 11) and agro-ecological features of Bangladesh (Fig. 12). A subtropical monsoon climate characterized by wide seasonal variations of rainfall (Fig. 11a), high relative humidity (Fig. 11b) and moderately warm temperatures (Fig. 11e) are the general climatic features of Bangladesh, with little variation across the country. Only the monsoon season (June to September) which accounts for over 71% of the annual rainfall. This compares to the relatively dry and cooler winters (December to February) which are usually rainless (only 2% annual rainfall). Minimum temperature of the winter season is around 10 °C (7-13 °C), however, the highest temperature of the summer occasionally exceeds 40 °C (Fig. 11f). Soil moisture quickly decreases in the pre-monsoon periods due to the combined effects of no rainfall, low humidity, high temperature, extended bright sunshine hours due to low cloud coverage and rising wind speed (Fig. 11).

Based on physiography, soil types, hydrology and agro-climatic features, Bangladesh is divided into 30 agro-ecological zones. However, due to year-round suitable agro-climatic conditions, rice is cultivated throughout the year with overlapping or short turnover periods; mainly in three seasons: aus (Mar-July), aman (May-Dec) and boro (Dec-June). Aman rice are of two types- broadcast aman and transplanted aman. Broadcast aman is a direct seeded traditional deepwater rice which matures after the monsoon period, i.e., mostly during Nov-Dec whereas transplanted aman is rainfed, sometimes irrigated. Aus and broadcast aman are cultivated in the upland and deepwater ecosystems, respectively. Boro rice is usually transplanted in Dec-Jan and is harvested before monsoon. Aman is the main rice cropping season since time immemorial in Bangladesh; the oldest Bengali literature, Charyapada only mentions aman rice. However, cultivation areas of broadcast aman and aus have been significantly decreased (over 80% and 60%, respectively) in the last four decades (Additional file 2: Figure S2). Broadcast aman (prolong flood tolerant rice) and aus (drought tolerant) are known for their stress tolerance capacity since they are grown during stress prone seasons. Boro cultivation area was sharply increased (over five-fold) in the last 5 decades (Additional file 2: Figure S2), mostly because of the expansion of irrigation facilities and the higher yield of modern varieties.

Flooding is the most prevalent and recurrent abiotic stress in Bangladesh due to its agro-ecological and geo-climatic features. The majority of the cultivable areas of Bangladesh are in floodplains and therefore flood-prone (Fig. 12a) with distinct types, intensities and depth (Fig. 12a, b). Millions of hectares of cultivable areas are in risk of either river flooding or flash floods. In addition, over 2 million hectares are in danger of tidal surges (Fig. 12a). Over 10% of the areas of the total flood-prone zones may experience a flooding depth over 1.8 m. The majority of the area of rabi and pre-kharif seasons are also drought-prone due to the combined effect of climatic (Fig. 11) and agro-ecological features of the pre-monsoon season (March to May). However, the severity of drought depends on the land types of particular region, their soil texture and moisture holding capacity, permeability, drainage and number of dry days etc. Apart from drought and flood, a few other constraints: salinity, alkaline and acid sulphate soils hamper rice cultivation and expansion in Bangladesh (Fig. 12d and Additional file 3: Figure S3). However, although the majority of the cultivable land are non-saline, hundreds of thousands of hectares of land, particularly in the costal belt areas (Additional file 3: Figure S3), suffer from salinity to various extents.

### Sparseness of Highly Abiotic Stress Tolerant Rice Accessions

We selectively analysed large-scale screening results that comprised over a thousand global rice accessions. Highly stress tolerant accessions are very rare; ranging from 0 to 5.3% (Table 2). Among all abiotic stresses, prolonged flood tolerant accessions are relatively common, i.e., over 5%. Deepwater rice adapts to rising flood water by rapid internode elongation to avoid submergence. Rapid internode elongation ability is a remnant feature of the wild progenitor (*O. rufipogon*). Anaerobic germination (AG) tolerance is the rarest among all abiotic stresses, i.e., only 6 accessions were highly tolerant among over 8000 germplasms. None of the accessions was found to be highly zinc deficiency tolerant in a zinc-deficient field screen. Similarly, none of the accessions was highly tolerant in the severe drought screening. Despite high sparseness, the majority of the highly tolerant accessions consistently originated from a very narrow geographic region, mostly within 20-24°N and 90-100°E (Table 2).

**Table 2:** Preferential Geographic Distribution Pattern of Abiotic Stress Tolerant Rice Accessions

Abiotic stress		Total number of accessions screened	Highly tolerant accessions^a^	Preferred latitude	Preferred longitude	Highly tolerant accessions (%)	No of highly tolerant accessions from Bangladesh	Highly tolerant accessions from Bangladesh (%)
Cold		6612	81	20-24°N	90-110°E	1.2	16	19.8
Salt		8004	31	20-24°N	90-100°E	0.4	26	83.9
Alkali		2649	83	20-24°N	90-100°E	3.1	48	57.8
Zinc	Field	960	0	20-24°N	90-100°E	0.0	(13)^b^	(28.3)^b^
	Greenhouse	119	3	20-24°N	80-90°E	2.5	NA	NA
Deepwater		1200	64	20-24°N	90-100°E	5.3	61	95.3
Flash flood		18,087	371	20-28°N	80-90°E	2.1	51	13.7
Flooded seed germination (AG)		8114	6	20-24°N	90-100°E	0.1	2	33.3
Drought - yield performance		988	66	20-24°N	90-100°E	5.3^c^	24	36.4
Drought -non-yield performance screening		38,433	na	na	na	0-37.6	2750	0-28.4

*na* not analysed as vegetative stage drought tolerance is not correlated with yield performance, *NA* no accession of Bangladesh screened

^a^List of highly tolerant accessions can be found in Additional file 4: Table S1

^b^Tolerant accessions were considered for rarity estimation, as no accession was found highly tolerant in the zinc-deficient field screening

^c^Although 6.7% accessions were found drought tolerant after field screening by yield performance, however, drought tolerant accessions were defined as any accessions that appeared in the top 25% in grain yield in any crop seasons (Torres et al. 2013). However, we excluded accessions of wet-season that were included in the top 25% in grain yield

Earlier we analysed preferential distribution pattern and population types of drought and flood tolerant rice accessions (Bin Rahman and Zhang 2016). However, in this review, we analysed all seven major abiotic stresses along with one nutrient deficiency tolerance. Surprisingly, Bangladesh has turned out to be the area of preference for highly tolerant accessions of all of the abiotic stresses along with one nutrient deficiency. In some cases, almost all of the highly tolerant accessions originated from Bangladesh or Bangladesh centred area (Table 2 and additional file 4: Table S1). Natural coincidence of the areas of preference of the 7 abiotic stresses along with one nutrient deficiency in a narrow geographic region is literally impossible; rather it is the specific pattern of preferential distribution of abiotic stress tolerant rice accessions. More surprisingly, Bangladesh is one of the most vulnerable climate change countries of the world where rice is literally the nutritional lifeline. On the average, rice accounts for nearly 70% of the calorific demand in Bangladesh (GRiSP 2013), where poor people basically live solely on rice. Bangladesh is the only rice-growing country where rice is represented in both the country’s national anthem and national emblem. Bangladesh is a relatively very small country (area only 147,570 km^2^); 65, 22 and 12 times smaller than the other top three rice producing countries, China, India, and Indonesia, respectively. However, Bangladesh is the 4th largest rice producer of the world (GRiSP 2013), where still hundreds of traditional rice varieties are cultivated (Hossain et al. 2013).

Aus rice varieties are well known for their abiotic stress tolerance capacity. Cultivation of aus rice is confined to Bangladesh and adjacent Indian states. The most primitive deepwater rice ecotype, Rayada, is often categorized as aus although some studies identified it as a distinct population type (Glaszmann 1987). No Bangladeshi accessions was screened in greenhouse screening for zinc deficiency tolerance, hence we see a gap in the longitude wise distribution (Fig. 3g arrow). However, if we fill the gap using the zinc deficiency field screening data pattern, then clearly it shows the same single peak preference and, as expected, it lies in Bangladesh. AG tolerant accessions are amongst the rarest of all abiotic stresses. However, from 6, 2 Bangladeshi rice accessions (Khaiyan, Kalongchi) are still highly tolerant. More interestingly, only two countries (India and Myanmar) share a border with Bangladesh where both of the countries also possess one AG tolerant rice accession each (India -Nanhi; Myanmar-Khao Hlan On). The largest QTL effect identified for AG tolerance, qAG-9-2 (also known as AG1), was identified from Myanmar landrace, Khao Hlan On (Angaji et al. 2010). Kretzschmar et al. (2015) identified a trehalose-6-phosphate phosphatase gene, OsTPP7, as the genetic determinant in qAG-9-2. Recently, the QTL was successfully introgressed into the elite cultivars/mega varieties like IR64, Ciherang to produce Sub1 + AG1 rice lines via MABC. Nearly 75% of drought tolerant accessions also originated from Bangladesh and adjacent Indian states. Likewise, PSTOL1 (Gamuyao et al. 2012) and SUB1A (Xu et al. 2006) were also identified from the landraces of the same region. Therefore, the preferential geographic distribution of abiotic stress tolerant rice accessions in Bangladesh or Bangladesh centred areas is undoubtedly the shared distribution pattern.

### Implication of the Specific Distribution Patterns of Abiotic Stress Tolerant Rice

We need to capitalize on the patterns for the development of abiotic stress tolerant rice varieties as well as the genetic and evolutionary bases of the preferential distribution pattern. Origin and domestication history of rice could help us to understand the specific distribution pattern of abiotic stress tolerant accessions. However, both origin and the place of rice domestication have long being highly debated topics. Vavilov (1926) considered India (Bangladesh was then part of India, became independent in 1971) as the place of rice domestication. A similar conclusion was drawn in later reports (Ramiah and Ghose 1951; Sampath and Govindaswamy 1958) based on the ecological similarities between wild and cultivated rice. However, some Chinese scientists have differed, and considered instead China to be the place of domestication as De Candolle postulated earlier (Oka 1988). Numerous small to large-scale studies have been carried out in the last couple of decades to put an end to these debates. Recently, based on the sequencing of thousands of accessions of wild and cultivated rice it was concluded that the Guangxi province of China as the most likely place of the first development of cultivated rice (Huang et al. 2012). However, the debate did not stop there as a re-analysis of the same dataset by a different group (Civáň et al. 2015) concluded multiple domestication centers of rice. Against this, the original authors argued that the reanalysis methods may be technically flawed (Huang and Han 2015). However, the preferential geographic distribution pattern of all major abiotic stress tolerant rice in Bangladesh or Bangladesh centred areas clearly suggest otherwise.

Recently, several agronomically important genes such as SUB1A (Xu et al. 2006), SNORKEL (Hattori et al. 2009), PSTOL1 (Gamuyao et al. 2012), DRO1 (Uga et al. 2013) have been identified from, FR13A, Gowai 38-9, Kasalath, Kinandang Patong, respectively. Except for Kinandang Patong, all other accessions also originated from the identified preferred Bangladesh centred areas/region. An earlier report considered Kinandang Patong as being a tropical japonica variety (Uga et al. 2008). However, recent sequencing-based population and genetic studies (Huang et al. 2012) confirmed it as a typical aus. Cultivation of aus rice is mostly confined to the identified preferred region. However, although the origin and evolution of cultivated rice are beyond the scope of this review, the preferential geographic distribution pattern of the highly tolerant accessions of all seven major abiotic stresses including cold along with one nutrient deficiency tolerance in a narrow geographic region of Bangladesh centred area clearly point to Bangladesh centred area as being the center of origin of *O. sativa*. We should perhaps ignore this interesting academic debate, and pay more attention to the shared distribution patterns of abiotic stress tolerant rice accessions as they can be utilized for the development of climate resilient varieties, as we cannot afford to fail the major global challenges like food security by 2050. The consequences could be catastrophic, costing millions of lives.

We know where exactly the abiotic stress tolerant accessions are clustered. We now have sufficient tools and techniques, e.g. genome sequencing, GWAS, automatic phenotypic platforms, even some sensor-based platforms that can measure field level data, to dig more deeply. More importantly, the genome sequencing costs have significantly decreased over the past decades. Unfortunately, only a few tolerant accessions of these abiotic stresses originating from Bangladesh have been sequenced. Therefore, the next immediate step should be genome sequencing of more highly tolerant rice accessions from Bangladesh to identify the signature of stress tolerance patterns by genome wide association studies. The success of GWAS in the pattern recognition of stress tolerance has already been established in numerous crop plants including rice (Huang et al. 2011).

We propose a concerted research effort to identify the genomic speciality of abiotic stress tolerant rice accessions and subsequent development of climate resilient rice varieties. **First,** the collection of the indigenous knowledge of stress tolerant rice accessions (locality and their special features known to farmers) in Bangladesh before they disappear. For instance, native farmers of Sylhet, Bangladesh call Kasalath (a well-known aus variety from which the PSTOL1 gene has been identified) as *Kasa Lota* (young green shoot). Kasa Lota is well known among the greater Sylhet rice farmers for its tolerance in the nutrient poor soil. **Second**, evaluation (both field and laboratory screening) of more landraces from Bangladesh for abiotic stress tolerances. **Third,** de novo assembly and deep sequencing of one or more traditional land races, particularly aus as we often experienced the absence of agronomically important genes in the rice reference genome, Nipponbare. For instance, Nipponbare completely lacks SNORKEL, PSTOL1 etc. alleles and possesses only intolerant alleles like SUB1A, DRO1. Therefore, it would not be unlikely to anticipate the absence of master regulator(s) of other stress tolerances in Nipponbare. **Fourth**, identification of genomic signatures of abiotic stress adaptations by both conventional and modern GWAS studies. However, we need more sophisticated bioinformatics and computational algorithms along with a cloud computing platform as the existing tools and methods are unable to handle the extraordinarily growing scale of data. **Fifth**, development of abiotic stress tolerant or climate-smart rice varieties through intense breeding efforts capitalizing the MABC strategy even before the identification of the detailed molecular mechanisms of tolerance. For instance, the identification of the genetic basis of the semi-dwarf trait (defective gibberellin 20-oxidase) (Spielmeyer et al. 2002) of the green revolutionary rice, IR8 occurred almost 40 years after the revolution of IR8 made, whereas Sub1A rice was literally developed (Neeraja et al. 2007) before the gene identification (Xu et al. 2006) as MABC required several years.

## Conclusion

Considering the impact of climate change on global food security and poverty, urgent concerted research efforts are necessary for the development of climate resilient varieties to cope with the impending population demand by utilizing the technological advancement, know-hows, and the preferential distribution pattern of abiotic stress tolerant rice.

## Additional files

Additional file 1: Figure S1. Geographic distribution pattern of salt tolerant rice accessions of a recent medium-scale screening. (a) Latitude-wise distribution, (b) Longitude-wide distribution, (c) Area of preference. (TIFF 80 kb)

Additional file 2: Figure S2. Rice cultivation area of Bangladesh. (TIFF 113 kb)

Additional file 3: Figure S3. Agroecologically constrained areas of Bangladesh (Map source: BARI, Bangladesh). (JPEG 362 kb)

Additional file 4: Table S1. List of abiotic stress tolerant rice accessions. (XLS 110 kb)
