# Supplementary figures and images for: Preferential Geographic Distribution Pattern of Abiotic Stress Tolerant Rice
Source: Rice (N Y). 2018 Feb 8;11:10. doi: 10.1186/s12284-018-0202-9 (PMC5805671; doi:10.1186/s12284-018-0202-9)

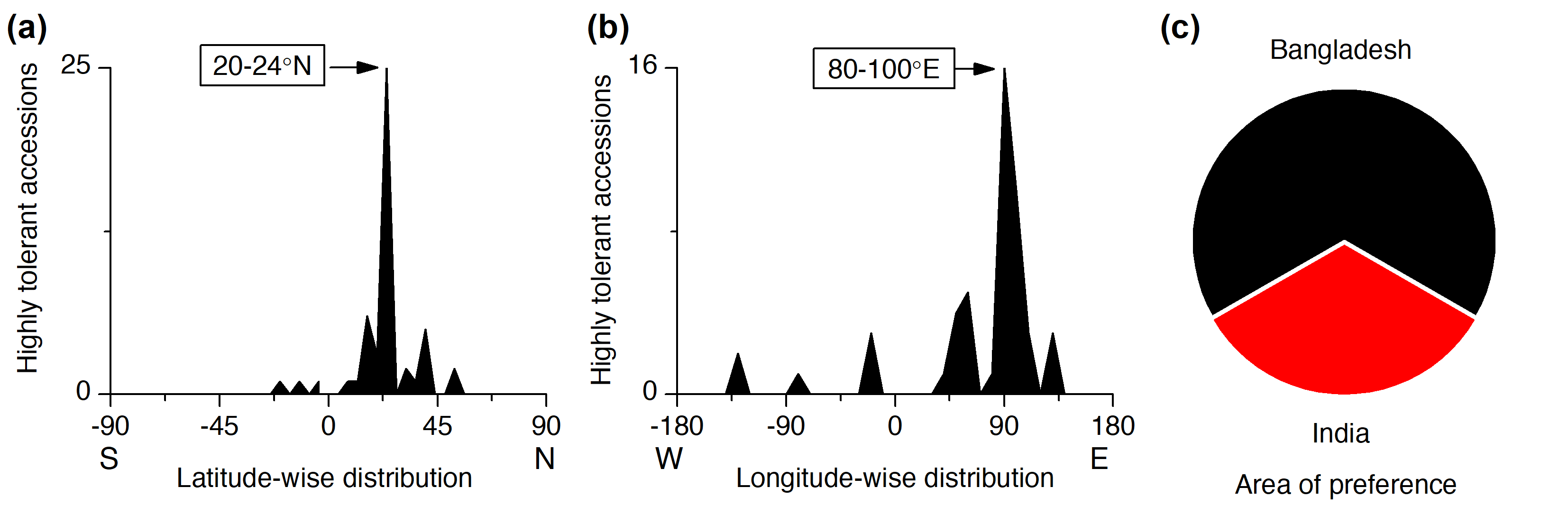

Supplement: Supplementary file 1 — Geographic distribution pattern of salt tolerant rice accessions of a recent medium-scale screening. (a) Latitude-wise distribution, (b) Longitude-wide distribution, (c) Area of preference. (TIFF 80 kb) [file 12284_2018_202_MOESM1_ESM.tif]

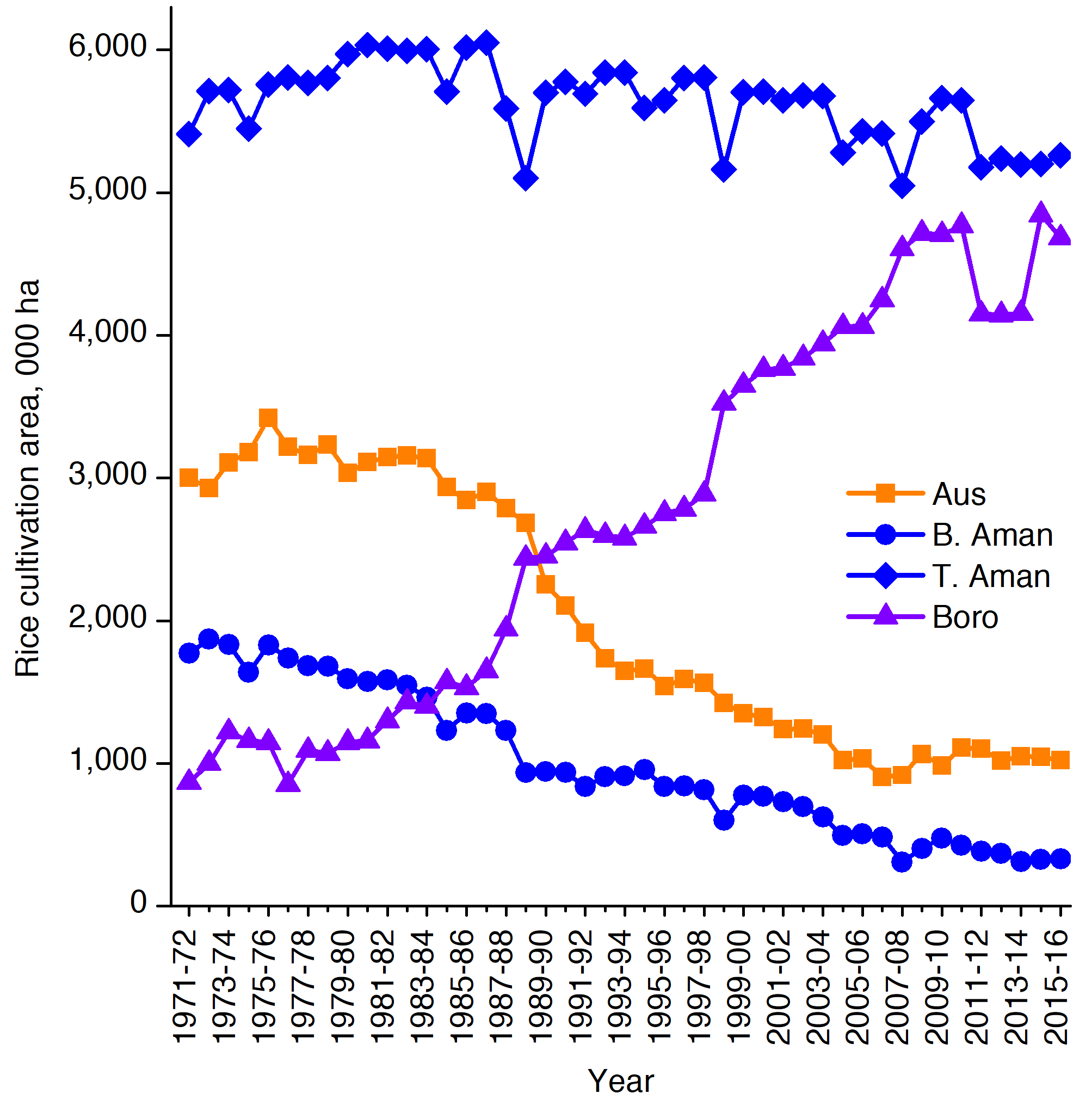

Supplement: Supplementary file 2 — Rice cultivation area of Bangladesh. (TIFF 113 kb) [file 12284_2018_202_MOESM2_ESM.tif]

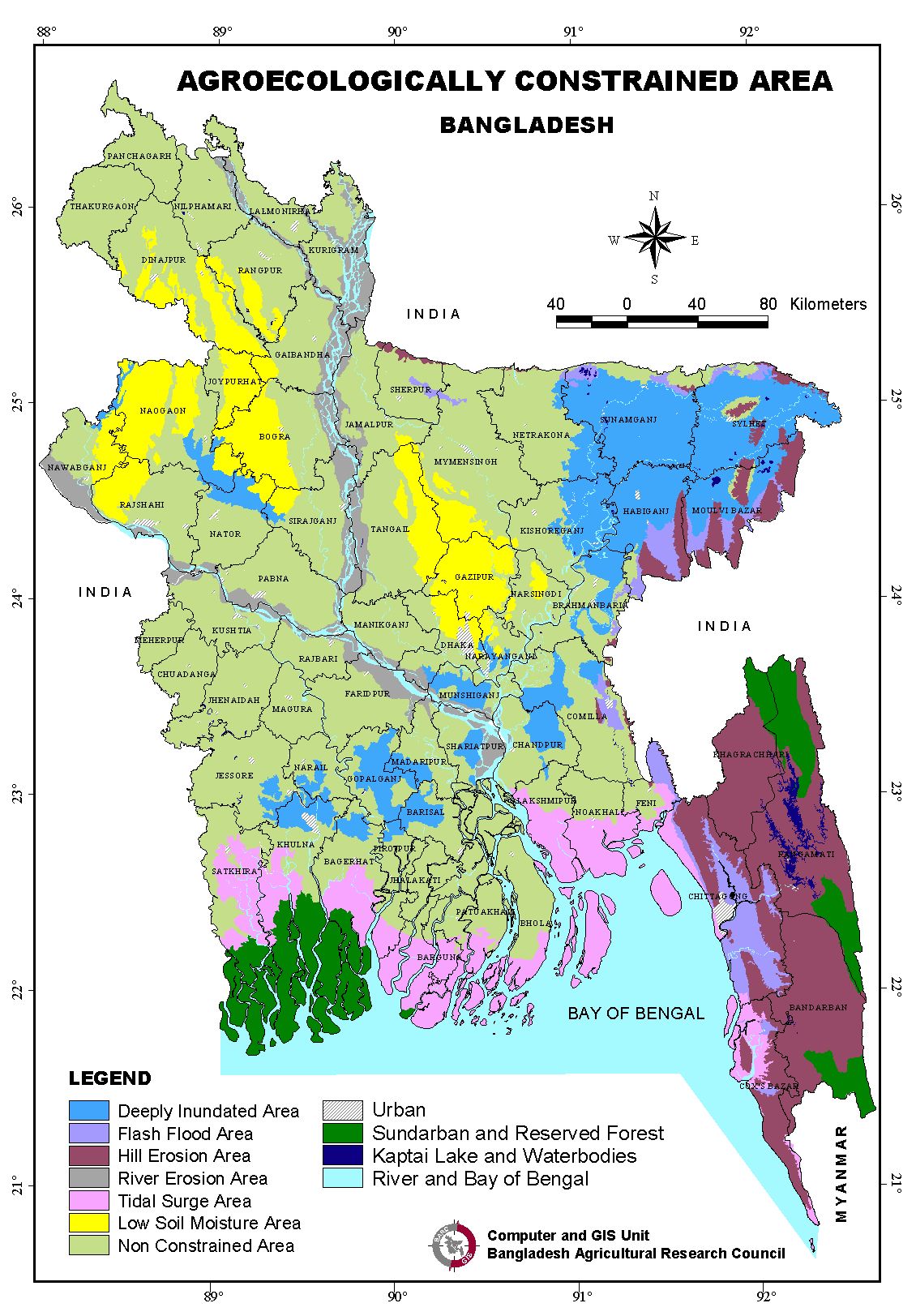

Supplement: Supplementary file 3 — Agroecologically constrained areas of Bangladesh (Map source: BARI, Bangladesh). (JPEG 362 kb) [file 12284_2018_202_MOESM3_ESM.jpg]
